# Demethylzeylasteral inhibits glioma growth by regulating the miR-30e-5p/MYBL2 axis

**DOI:** 10.1038/s41419-018-1086-8

**Published:** 2018-10-10

**Authors:** Kui Zhang, Gang Fu, Guangzhao Pan, Chongyang Li, Li Shen, Renjian Hu, Shunqin Zhu, Yibiao Chen, Hongjuan Cui

**Affiliations:** 1 State Key Laboratory of Silkworm Genome Biology, The Institute of Sericulture and Systems Biology, Southwest University, 400716 Chongqing, China; 2 Chongqing Engineering and Technology Research Center for Silk Biomaterials and Regenerative Medicine, 400716 Chongqing, China; 3 Southwest University Engineering Research Center for Cancer Biomedical and Translational Medicine, 400715 Chongqing, China; 4 College Pharmacy and Biological Engineering, Chongqing University of Technology, 400716 Chongqing, China

## Abstract

Glioma is the most common and malignant form of primary brain tumour, and is characterised by high proliferation and extensive invasion and neurological destruction. Demethylzeylasteral (T-96), which is extracted from *Tripterygium wilfordii*, is considered to have immunosuppressive, anti-inflammatory and anti-angiogenic effects. Here, the anti-tumour effect of T-96 on glioma was evaluated. Our results demonstrated that T-96 significantly inhibited glioma cell growth and induced cell cycle arrest in G1 phase but did not induce apoptosis. Cell invasion and migration were dramatically suppressed after treatment with T-96. Almost all genes related to cell cycle and DNA replication were downregulated after treatment with T-96. Our results showed that miR-30e-5p was noticeably upregulated after T-96 treatment, and MYBL2, which is involved in cell cycle progression and is a target gene of miR-30e-5p, was significantly reduced in synchrony. Overexpression of MYBL2 partially rescued the T-96-induced inhibition of cell growth and proliferation. Moreover, a miR-30e-5p antagomir significantly reduced the upregulation of miR-30e-5p expression induced by T-96, leading to recovery of MYBL2 expression, and partially rescued the T-96-induced inhibition of cell growth and proliferation. More important, T-96 effectively upregulated miR-30e-5p expression and downregulated MYBL2 expression, thus inhibiting LN-229 cell tumour growth in a mouse model. These results indicated that T-96 might inhibit glioma cell growth by regulating the miR-30e-5p/MYBL2 axis. Our study demonstrated that T-96 might act as a promising agent for malignant glioma therapy.

## Introduction

Glioma is the most common and lethal type of primary brain tumour, with a poor survival outcome, and is characterised by high proliferation, invasion and metastasis ability^1–5^. Investigations have shown that the incidence of glioma increases by 3% per year^6^. Surgery followed by radiotherapy and temozolomide (TMZ) chemotherapy are currently the main therapeutic strategies for glioma^3^. Progress has been made in the diagnosis and treatment of glioma, but prognosis remains relatively poor^7^. Therefore, there is a pressing need for more effective therapeutic strategies for malignant glioma therapy.

Tripterygium wilfordii Hook F (TwHF) is commonly known as “lei gong teng” or “thunder god vine,” and is widely distributed in China, Korea and Japan^8^. In the past half century, TwHF has been widely applied in the treatment of autoimmune and inflammatory diseases, including rheumatoid arthritis^9,10^, nephritis^11^, ankylosing spondylitis^12^, systemic lupus erythaematous and psoriasis^13^. Many studies have shown that the active ingredient extracts of TwHF also exhibit anti-tumour activities. Wang Z and colleagues found that TwHF seems to be able to sensitise resistant prostate cancer cells to the chemotherapeutic effect of docetaxel^14^.

A variety of bioactive compounds, including diterpenoids, triterpenoids, alkaloids and other small molecules, have been isolated from TwHF and characterised^15^. In numerous compounds, triptolide is the most investigated class of bioactive TwHF compounds. It has been reported that triptolide can induce apoptosis in various human tumour cells, including leukaemia^16^, cholangiocarcinoma^17^, gastric cancer^18^, myeloma^19^, pancreatic carcinoma^20,21^ and oral cancer cells^22^. Celastrol has also been widely studied and was found to inhibit tumour growth^23,24^, and suppress invasion^25^ and metastasis^26^.

Demethylzeylasteral (T-96) is a triterpene compound extracted from TwHF. The pharmacological toxicity of T-96 is relatively lower than that of other bioactive compounds, such as triptonide, celastrol and triptolide. Previous studies have demonstrated that T-96 has immunosuppressive effects and can be used in animal transplantation models^27–29^. Recent studies indicated that T-96 improves lupus nephritis by inhibiting NF-κB pathway activation in mice^30^. In addition, it has been shown that T-96 can inhibit angiogenesis, tumour cell proliferation and tumour growth^31^. Recent studies have shown that T-96 inhibits pancreatic cancer cell proliferation and induces cell apoptosis and autophagy. T-96 also enhanced the chemosensitivity of pancreatic cancer cells to gemcitabine^32^. Our previous studies suggested that T-96 could inhibit melanoma cell proliferation and induce apoptosis by suppressing the expression of MCL1^33^. Other studies have shown that T-96 is a potential therapeutic agent for developing novel therapeutic strategies for human cancer. However, the anti-tumour effect of T-96 and the underlying regulation mechanisms need to be further explored.

In the present study, the anti-tumour activity of T-96 was evaluated in glioma. Our work is the first to reveal that T-96 inhibits glioma growth by inhibiting DNA replication and causes G1/S cell cycle arrest by downregulating MYBL2. Our study suggests that T-96 might be a potential candidate agent for malignant glioma therapy.

## Results

### T-96 inhibits glioma cell growth and proliferation

**Fig. 1:**
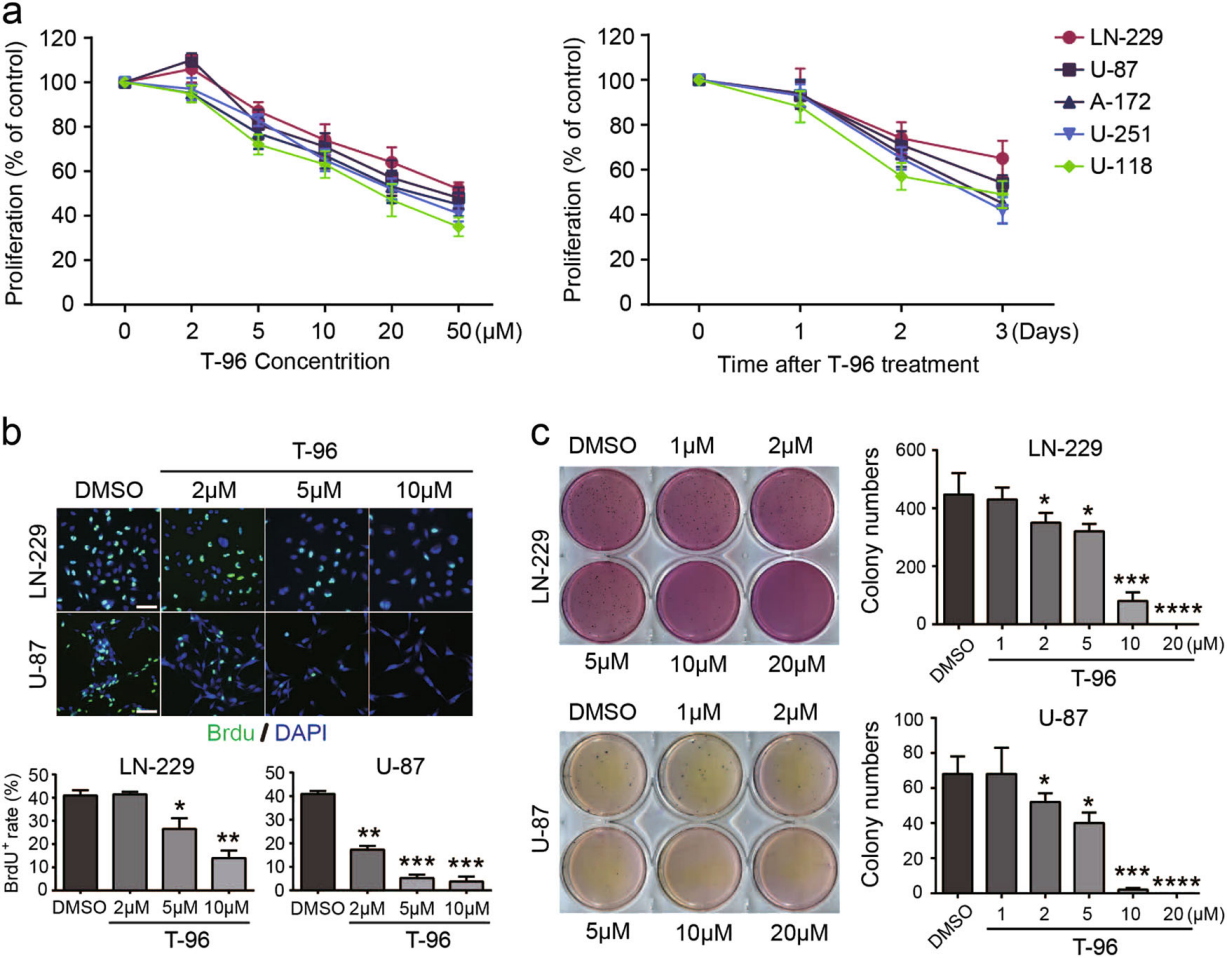
T-96 inhibited cell growth and proliferation in human glioma cells. **a** Concentration- and time-dependent effects of T-96 on glioma cells, including LN-229, U-87, A-172, U-251 and U-118 cells. Left, glioma cells were treated with different concentrations of T-96 for 2 days; right, cells were treated with 10 μM T-96 for different times. Survival was evaluated using MTT assays, and the data are presented as the means ± SD, *n* = 3). **b** Images of LN-229 and U-87 cells positive for BrdU staining after treatment with DMSO or the indicated concentration of T-96. Scale bar = 100 μm. The histogram demonstrates the results of the quantification of the number of BrdU-positive cells in LN-229 and U-87 cells. **c** Soft agar assays were used to investigate the colony formation abilities of LN-229 and U-87 cells after treatment with DMSO or the indicated concentrations of T-96. All data were analysed using unpaired Student’s t-tests and are shown as the means ± SD. *p < 0.05, **p < 0.01, ***p < 0.001, ****p < 0.0001

To investigate the effect of T-96 on cell viability, glioma cells, including LN-229, U-87, A-172, U-251 and U-118 cells, were used to examine the effect of T-96 on tumour cell line growth. When treated with different concentrations of T-96, the proliferation of all tested cells was significantly decreased in a dose-dependent manner. All the detected cell lines were then treated with 10 μM T-96, and the cell proliferation rates were significantly inhibited in a time-dependent manner (Fig. 1a). These results demonstrated that T-96 has a broad-spectrum anti-tumour effect on human glioma cells. According to microscopy analyses, cell numbers were decreased after treatment with different concentrations of T-96 compared with the control group (Fig. S1a). The cell viability was measured with MTT assays, which revealed that T-96 significantly inhibited cell growth in a dose-dependent manner (Fig. S1b, c). A BrdU staining assay showed that T-96 significantly decreased the percentage of BrdU-positive cells compared with the control in a dose-dependent manner (Fig. 1b). Soft agar assays were performed to further investigate the effect of T-96 on the self-renewal capability of glioma cells. The results showed that the colonies were smaller and fewer in number in the T-96-treated cells compared with the control cells (Fig. 1c). These results suggest that T-96 remarkably inhibited human glioma cell growth and proliferation.

### T-96 inhibits cell growth by inducing cell cycle arrest, but not apoptosis in glioma cells

**Fig. 2:**
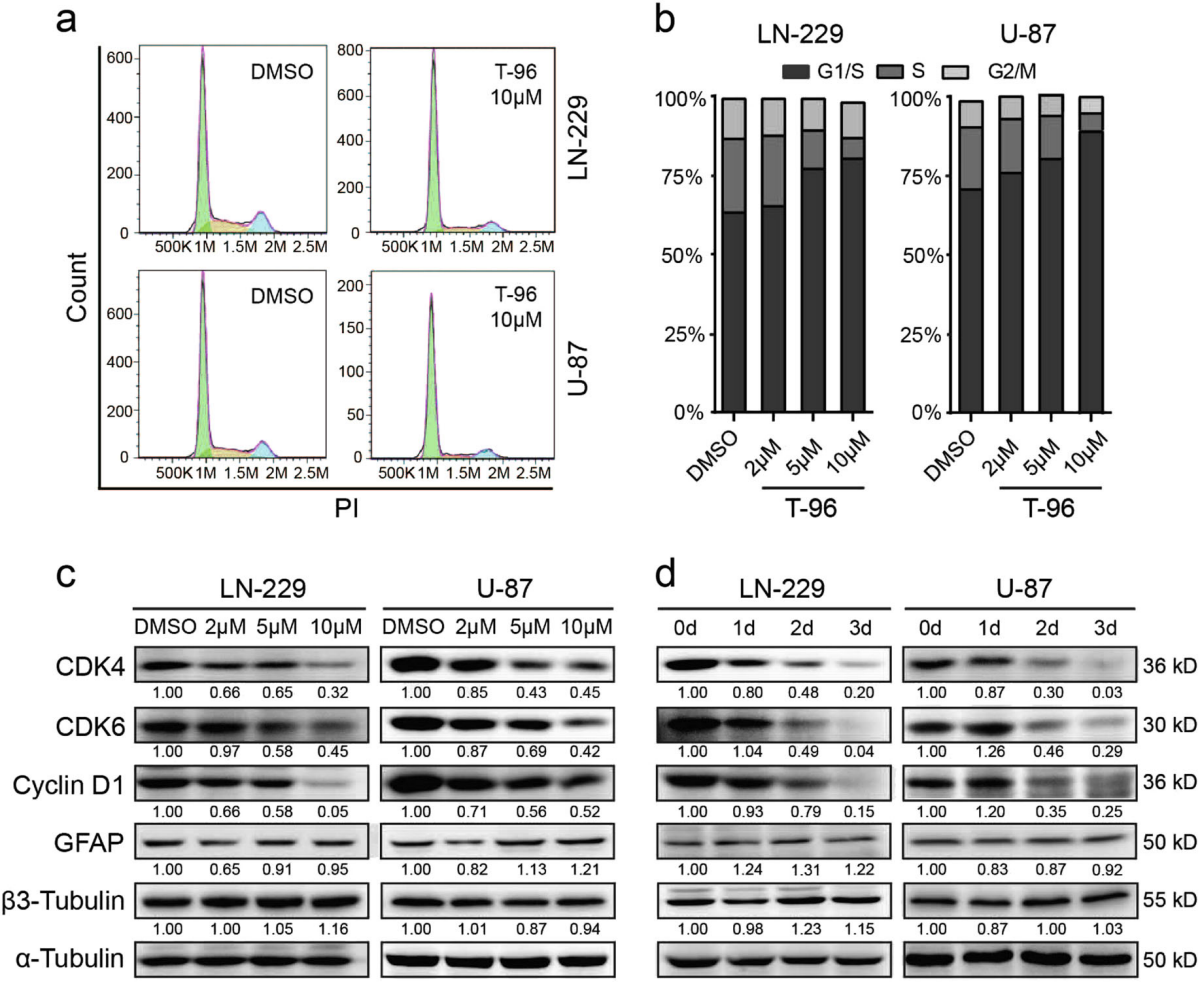
T-96 caused G1/S cell cycle arrest in human glioma cells. **a**, **b** The cell cycles of LN-229 and U-87 cells were investigated via flow cytometry after treatment with DMSO or the indicated concentration of T-96 for 2 days. **c**, **d** Western blotting assays were performed to detect the expression of CDK4, CDK6, Cyclin D1, GFAP, β3-Tubulin and cyclin D1 in LN-229 and U-87 cells after treatment with T-96

To explore the mechanism underlying T-96 inhibition of cell growth and proliferation, the effect of T-96 on the cell cycle was examined via flow cytometry. In LN-229 and U-87 cells, there was an approximately 30% increase in the number of cells in G1 phase among the 10 μM T-96-treated cells compared with control cells (Fig. 2a). The results revealed that T-96 treatment caused significant accumulation of cells in the G1/S phase in a dose-dependent manner (Fig. 2b). These results suggested that T-96 inhibits cell growth and proliferation by inducing cell cycle arrest at the G1 phase. To further confirm the results, the expression levels of cyclin-dependent kinase (CDK) 4, CDK6 and cyclin D1, which can promote cell passage through the G1/S checkpoint, were analysed by Western blot. The results revealed that the expression levels of the examined proteins were significantly reduced in T-96-treated cells in a dose-dependent and time-dependent manner. Cell proliferation and differentiation show a remarkable inverse relationship, and stimuli that promote differentiation may trigger pathways that induce cell cycle arrest at G0/G1. Therefore, two differentiation markers, GFAP and β3-tubulin, were also detected, and the results showed that the expression of these proteins was not significantly different compared with that in control cells (Fig. 2c, d). These results suggest that T-96 induced cell cycle arrest at the G1 phase by inhibiting the expression of related CDKs and cyclins.

No significant apoptosis was observed in T-96-treated cells during the experiment according to microscopy analysis. To confirm whether the reduced cell viability was caused by apoptosis, the apoptosis rate was determined by flow cytometry, as described in the Methods section. As shown in Fig. S2a, no obvious apoptosis appeared after T-96 treatment. Western blot assays were conducted to confirm this finding, and the results showed that the cleaved fragment of caspase-3 was not detected after treatment with T-96 in both LN-229 and U-87 cells (Fig. S2b, c). Cell senescence was also detected, and no signal was observed in the cells after treatment with T-96 (Fig. S3). These results suggest that T-96 does not induce apoptosis or senescence in glioma cells.

### T-96 inhibited cell migration and invasion in glioma cells

**Fig. 3:**
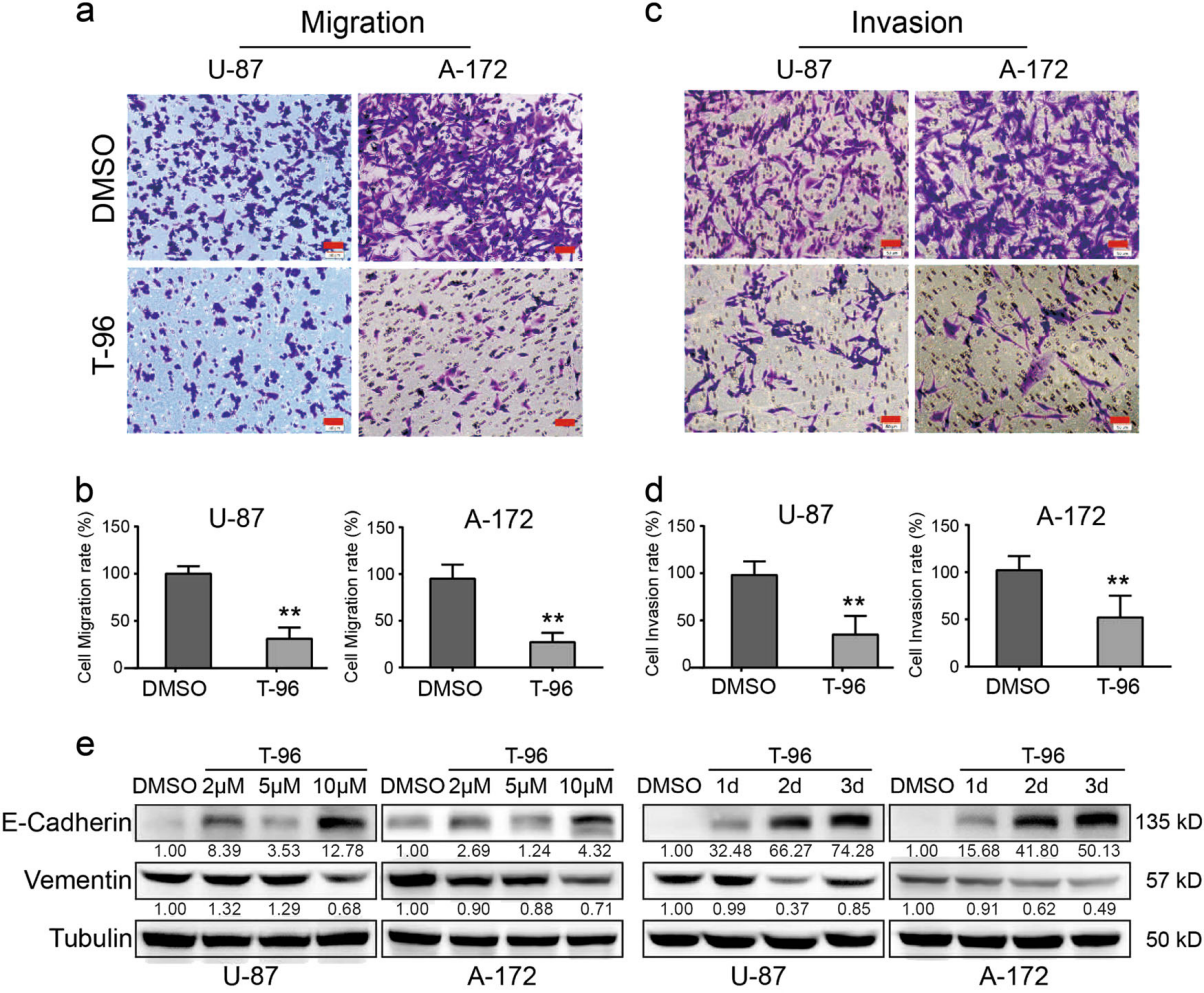
T-96 inhibited cell migration and invasion in human glioma cells. **a** Transwell migration assays of U-87 and A-172 cells treated with DMSO or 10 μM T-96 for 1 day. **b** The statistical analysis is presented in histograms, and the migration rates were normalised by the proliferation rate. **c** Transwell invasion assays of U-87 and A-172 cells treated with DMSO or 10 μM T-96 for 1 day. **d** The statistical analysis is presented in histograms, and the invasion rates were normalised by the proliferation rate. **e** The expression of E-cadherin and vimentin were analysed by Western blot. Tubulin was used as the control. All data were analysed using unpaired Student’s t-tests and are shown as the means ± SD. **p < 0.01

The effects of T-96-treatment on the migration and invasion abilities of human glioma cells were also evaluated via transwell migration and invasion assays. LN-229, U-87 and A-172 cells were used in these assays, and similar results were obtained. Here, data from U-87 and A-172 cells are shown. Migration assays demonstrated that the cell migration ability of the cells treated with T-96 was significantly affected compared with the control cells (Fig. 3a). The cell migration rates of U-87 and A-172 cells were summarised, and the results showed that T-96-treated cells exhibited obvious inhibition of cell migration compared with cell treated with the control dimethyl sulfoxide (DMSO) (Fig. 3b). The results of invasion assays showed that the number of cells that penetrated through the Matrigel-coated membrane was significantly reduced after treatment with T-96 compared with control cells (Fig. 3c). The statistical results are shown as representative histograms and are consistent with the above-described findings (Fig. 3d). The expression levels of E-cadherin and vimentin were evaluated with Western blot assays. The results showed that E-cadherin expression was significantly upregulated after treatment with T-96 in a dose-dependent and time-dependent manner. However, vimentin expression was remarkably downregulated (Fig. 3e). These results suggested that T-96 treatment reversed epithelial-mesenchymal transition (EMT) of malignant glioma cells. Therefore, our results indicate that T-96 can strongly inhibit the cell migration and invasion abilities of glioma cells.

### T-96 downregulated genes related to DNA replication and cell cycle progression

**Fig. 4:**
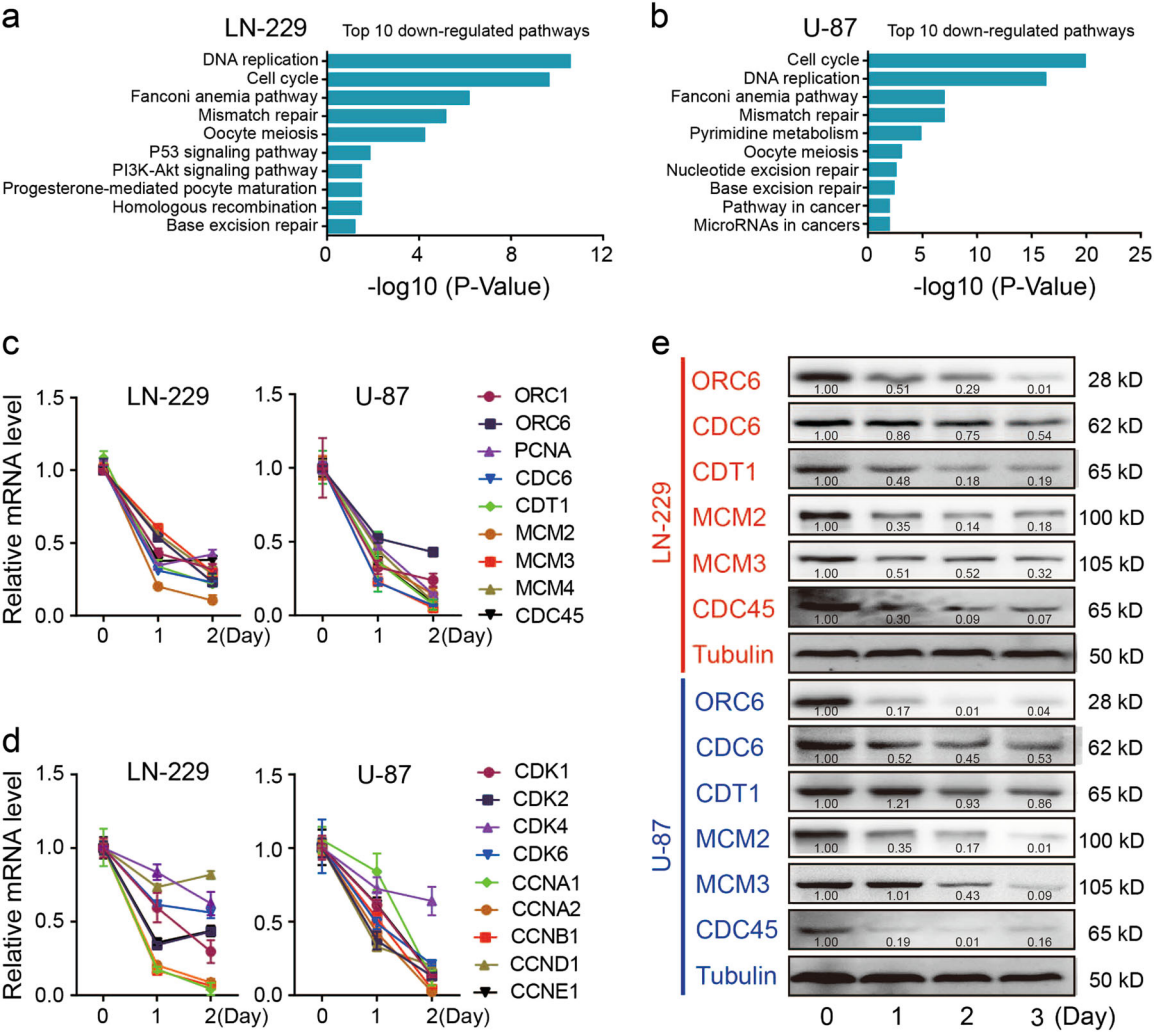
T-96 inhibits the expression of genes related to cell cycle progression and DNA replication. **a**, **b** GO analysis of genes downregulated (determined via whole transcriptome analyses (RNA-seq)) in LN-229 or U-87 cells after treament with T-96. The top 10 biological process terms based on fold enrichment are shown. **c**, **d** Real-time quantitative PCR analyses of cell cycle-related and DNA replication-related genes in LN-229 and U-87 cells after treatment with or without 10 μM T-96 for the indicated time. **e** Western blot analyses of several components of the replication complex and associated regulators, including ORC6, CDC6, CDT1, MCMs and CDC45. Tubulin was used as the control

To investigate how T-96 inhibits cell growth and proliferation, transcriptome analyses were performed on T-96-treated and DMSO-treated cells. Gene ontology (GO) analysis revealed that genes downregulated by T-96 treatment were significantly enriched for GO terms associated with DNA replication and cell cycle progression (Fig. 4a, b). KEGG results indicated that these genes were widely downregulated (Fig. S4). These differentially expressed genes were validated by qRT-PCR and Western blot. These results are consistent with the transcriptome data (Fig. 4c–e).

### Overexpression of MYBL2 rescued T-96-induced cell growth inhibition in glioma cells

**Fig. 5:**
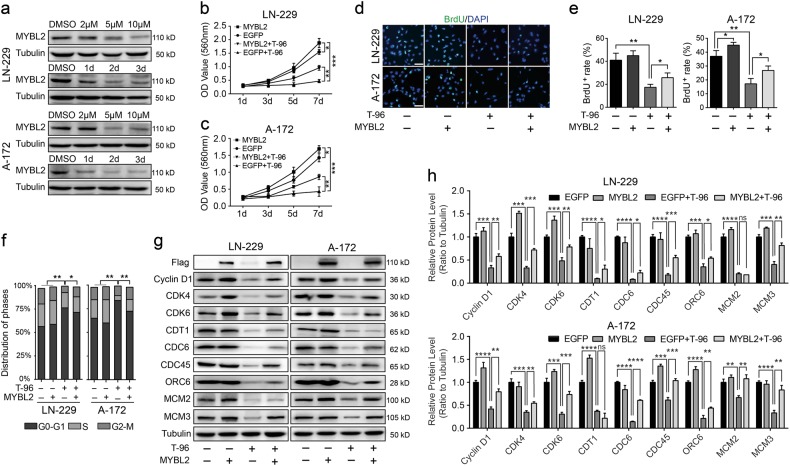
MYBL2 is required for T-96-induced cell growth and proliferation inhibition. **a** MYBL2 expression was analysed via Western blot assays after cells were treated with T-96. Cells were treated with the indicated concentration of T-96 for the indicated times. In the concentration gradient group, cells were treated with different concentrations of T-96 (2, 5 and 10 μM) for two days, and DMSO was used as the control; in the time gradient group, cells were treated with 10 μM T-96 for different times (1, 2 and 3 days), and DMSO was used as the control. **b**, **c** The effect of DMSO or T-96 on the viability of MYBL2/EGFP-overexpressing LN-229 and A-172 cells. **d** Representative image of BrdU staining of MYBL2-overexpressing or EGFP-overexpressing LN-229 and A-172 cells after treatment with DMSO or 10 μM T-96 for 2 days; scale bar = 100 μm. **e** Quantification of BrdU-positive staining rates in MYBL2-overexpressing or EGFP-overexpressing LN-229 and A-172 cells after treatment with DMSO or 10 μM T-96 for 2 days. **f** Cell cycle distribution was analysed in MYBL2-overexpressing or EGFP-overexpressing LN-229 and A-172 cells after treatment with DMSO or T-96 for 2 days. **g** Western blot assays were performed to evaluate proteins related to the cell cycle and DNA replication in MYBL2-overexpressing or EGFP-overexpressing LN-229 or A-172 cells after treatment with DMSO or T-96 for 2 days. Tubulin was used as the control. **h** Densitometry of Western blot in the panel **g**. All data were analysed using unpaired Student’s *t*-tests and are shown as the means ± SD. *p < 0.05, **p < 0.01, ***p < 0.001, ****p < 0.0001; ns no significant difference

Analysis of the transcriptome data revealed that the transcription factor MYBL2 was downregulated by 3.06-fold and 3.05-fold after treatment with T-96 (data not shown). The expression level of MYBL2 in different glioma cell lines was detected, and the results showed that MYBL2 is widely expressed in all the examined cell lines, with the greatest expression in U-87 cells(Fig. S5). The results of qRT-PCR and Western blot assays revealed that the expression of MYBL2 was significantly downregulated in a dose-dependent and time-dependent manner (Fig. S6 and Fig. 5a). These results indicated that MYBL2 may play a key role in T-96-induced cell growth and proliferation inhibition. A follow-up, rescue experiment was conducted to determine the role that MYBL2 played. LN-229 and A-172 cells were infected with lentiviruses encoding the human MYBL2 gene, and enhanced green fluorescent protein (EGFP) was used as the control. MTT assays were conducted to investigate the cell growth curve for one week after the addition of T-96 or DMSO in MYBL2/EGFP-overexpressing LN-229 (Fig. 5b) and A-172 cells (Fig. 5c). The results indicated that MYBL2 accelerated the growth of cells. Furthermore, overexpression of MYBL2 significantly reduced the cell growth inhibition induced by T-96 in both LN-229 and A-172 cells. BrdU assays were used to detect cell proliferation. The results suggested that the cell proliferation ability was partially rescued after MYBL2 overexpression in T-96-treated cells compared with in T-96-treated EGFP-overexpressing cells (Fig. 5d, e). The results suggested that overexpressing MYBL2 promoted cell growth and proliferation and significantly reduced the cell growth inhibition induced by T-96 (*p* < 0.01). In summary, these results demonstrate that T-96-induced inhibition of proliferation was rescued by overexpression of MYBL2 in glioma cells.

### MYBL2 overexpression rescues T-96-induced cell cycle arrest in glioma cells

Our results show that overexpression of MYBL2 rescued the inhibitory effects of T-96 on cell growth in glioma cells. It was speculated that T-96 might arrest the cell cycle by downregulating MYBL2 in glioma cells. Cell cycle was analysed via flow cytometry in MYBL2- and EGFP-overexpressing cells that were treated with T-96 or DMSO (Fig. 5f). Overexpression of MYBL2 upregulated the expression of genes related to cell cycle and DNA replication, including cyclin D1, CDKs, ORC6, CDC6, CDT1, MCMs and CDC45. Furthermore, the expression levels of the abovementioned proteins were higher in T-96-treated MYBL2-overexpressing cells than in T-96-treated EGFP-overexpressing cells (Fig. 5g, h). These data demonstrate that MYBL2 overexpression rescued T-96-induced cell cycle arrest in glioma cells.

### T-96 downregulated MYBL2 by upregulating miR-30e-5p

**Fig. 6:**
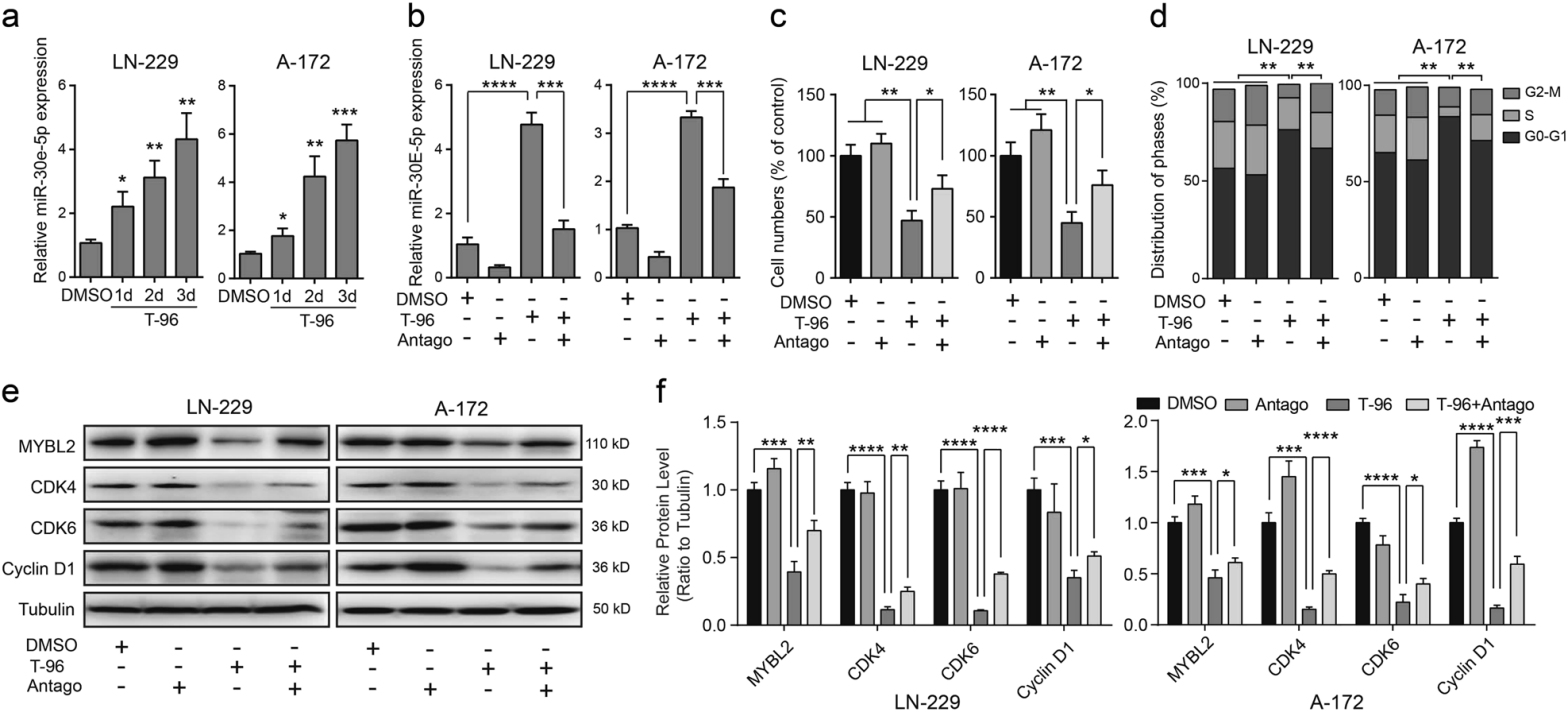
The miR-30e-5p antagomir (Antago) blocked the effects induced by T-96 in glioma cells. **a** Quantity real-time PCR (qRT-PCR) assays were performed to evaluate the expression of miR-30e-5p after treatment of LN-229 and A-172 cells with DMSO or 10 μM T-96 for the indicated time. **b** The expression of miR-30e-5p after T-96 treatment or T-96 and the miR-30e-5p antagomir treatment for 2 days. DMSO was used as the control. **c** LN-229 and A-172 cells were treated with DMSO, 10 μM T-96, the miR-30e-5p antagomir, or T-96 and the miR-30e-5p antagomir for 2 days, and the cell viability was evaluated with MTT assays. **d** LN-229 and A-172 cells were treated with DMSO, the miR-30e-5p antagomir, 10 μM T-96 or T-96 and the miR-30e-5p antagomir for 2 days, and cell cycle was analysed via flow cytometry. **e** Western blot assays were used to detect the expression of MYBL2, CDK4, CDK6 and cyclin D1 after treatment with DMSO, the miR-30e-5p antagomir, 10 μM T-96, or T-96 and the miR-30e-5p antagomir for 2 days. **f** Densitometry of Western blot in the panel **e**. All data were analysed using unpaired Student’s t-tests and are shown as the means ± SD. *p < 0.05, **p < 0.01, ***p < 0.001, ****p < 0.0001; ns no significant difference

In MYBL2-overexpressing cells, exogenous MYBL2 proteins were not significantly decreased after treatment with T-96 compared with DMSO, especially in LN-229 cells (Fig. 5g). Therefore, we speculated that T-96 suppresses MYBL2 expression at the transcriptional level. As a class of small non-protein-coding RNAs, microRNAs (miRNAs) can negatively regulate the expression of target genes at the transcriptional level. All miRNAs that could theoretically target the 3’UTR of *MYBL2* were analysed, and miR-30e-5p was found to be significantly upregulated in all detected cells (Fig. S7). miR-30e-5p, which can target MYBL2^34^, was significantly upregulated after treatment with T-96 compared with controls in a time-dependent manner, both in LN-229 and A-172 cells (Fig. 6a). We hypothesised that T-96 might inhibit cell proliferation by regulating the miR-30e-5p/MYBL2 axis. To confirm this, a miR-30e-5p antagomir (Antago) was applied. Real-time PCR assays showed that the expression of miR-30e-5p was significantly decreased in antagomir-treated cells compared with cells treated with T-96 alone (Fig. 6b). The results demonstrated that the increase in miR-30e-5p after cells treatment with T-96 was successfully blocked by the miR-30e-5p antagomir. The proliferation of LN-229 and A-172 cells treated with DMSO, the miR-30e-5p antagomir, T-96 or T-96 together with the miR-30e-5p antagomir was investigated using MTT assays. The results indicated that downregulation of miR-30e-5p expression in T-96-treated cells partially rescued the cell survival rate (Fig. 6c). In addition, downregulation of miR-30e-5p expression blocked the cell cycle arrest induced by T-96 in LN-229 and A-172 cells (Fig. 6d). Western blot assays suggested that the antagomir increased the MYBL2 expression in T-96-treated cells. Additionally, the antagomir of miR-30e-5p also increased the expression levels of CDK4, CDK6 and cyclin D1 compared with cells treated with T-96 alone (Fig. 6e).

### T-96 inhibits tumour growth in vivo

**Fig. 7:**
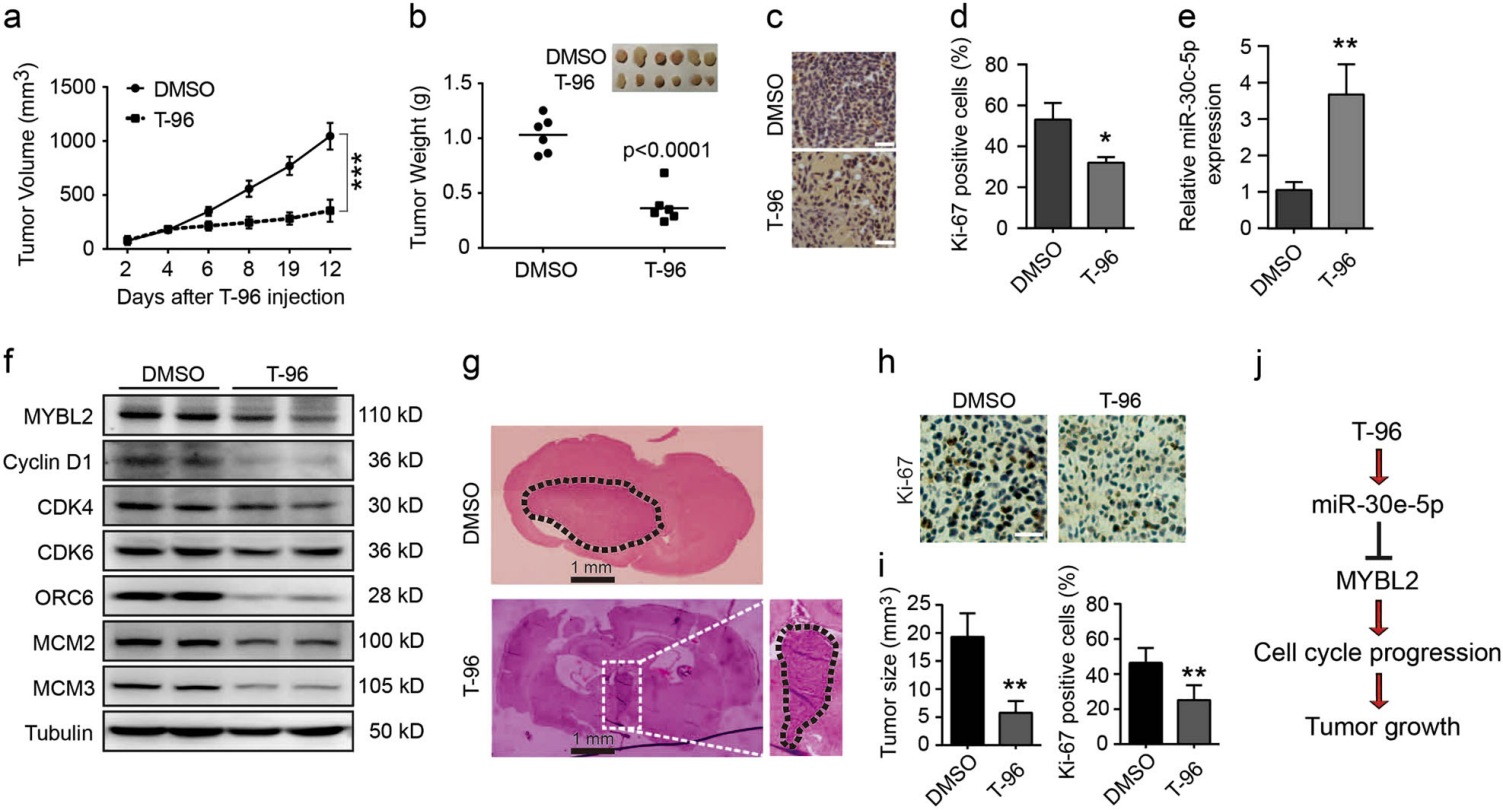
The anti-tumour effect of T-96 was evaluated in vivo. **a**, **b** Human LN-229 cells were subcutaneously injected into the right flanks of female nude mice. When tumours reached a certain volume, T-96 (30 mg/kg) was injected once every two days, and DMSO injections were administered as the control. Two days after the first injection, tumour volume was measured every 2 days. Two days after the sixth injection, the mice were killed, and tumour weight was measured. **c** Heamatoxylin and eosin (H&E) staining of the indicated xenograft tumours. **d** Effect of T-96 on cell proliferation in subcutaneously transplanted tumours derived from LN-229 cells. **e** qRT-PCR was performed to evaluate the expression of miR-30e-5p in tumour tissues treated with DMSO or T-96. **f** Western blot assays were performed to evaluate the expression of MYBL2, cyclin D1, CDK4, CDK6, ORC6, MCM2 and MCM3 in tumour tissues treated with DMSO or T-96. Tubulin was used as the control. **g** Orthotopic implantation was performed with LN-229 cells, and representative images of the heamatoxylin and eosin (H&E) staining are presented; scale bar = 1 mm. **h** Immunohistochemical staining for Ki67 in tumour tissues. **i** Effects of T-96 on tumour size (left) and LN-229 cell proliferation (right). **j** Diagram of the mechanism by which T-96 inhibits cell proliferation and tumour progression in human glioma cells. All data were analysed using unpaired Student’s *t*-tests and are shown as the means ± SD. *p < 0.05, **p < 0.01, ***p < 0.001

To further investigate the effects of T-96 in vivo, LN-229 cells were transplanted subcutaneously into nude mice, as described in our previous study^35^. When the tumours reached a certain volume, T-96 (30 mg/kg) was injected 6 times every 2 days, and an equivalent amount of DMSO was used as the control. Tumour growth and mouse weight were measured every two days. The weight of the mice did not differ between the DMSO and T-96 treatment groups (Fig. S8), indicating that the drug was safe in mice. The tumour volume and weight were significantly decreased in mice after treatment with T-96 compared with control mice (Fig. 7a, b). The results indicated that T-96 treatment remarkably inhibited tumour growth in vivo. To investigate whether T-96 inhibited the tumour growth of LN-229 cells by inhibiting cell proliferation, Ki67 was detected via immunohistochemistry. As shown in Fig. d, Ki67 expression in tumour tissues was significantly decreased after treatment with T-96 compared with controls. Real-time PCR was performed to detect the expression of miR-30e-5p. Consistent with the cell experiments, miR-30e-5p expression was markedly increased in tumours treated with T-96 compared with controls (Fig. 7e). Western blot assays indicated that MYBL2 expression levels were significantly decreased (Fig. 7f). Furthermore, cell cycle-related proteins, including cyclin D1, CDKs, ORC6 and MCMs, were downregulated in the xenograft tumour tissues after treatment with T-96 compared with controls (Fig. 7f), and these trends were largely consistent with the previous experimental results. These results demonstrated that T-96 might also inhibit glioma cell growth by blocking normal cell cycle progression in tumour xenograft mice. To further investigate the anti-tumour activity of T-96 in vivo, orthotopic implantation experiments were performed to investigate whether T-96 can pass through the blood-brain barrier, which is a main issue for the treatment of gliomas, and successfully reach the desired focus. Our results indicate that T-96 significantly reduced the size and proliferation of transplanted tumours in mouse brains compared with tumours in the control mice (Fig. 7g–i). In summary, we hypothesise that T-96 suppressed tumour growth by regulating the miR-30e-5p/MYBL2 axis in glioma (Fig. 7j).

## Discussion

Glioma is the most common and lethal primary intracranial tumour, with an incidence of 5/100,000 individuals^36^. Over the past decades, several agents, such as temozolomide (TMZ) and bevacizumab, have been used in clinical glioma therapy and have effectively improved the living quality of patients^3,37^. However, although great advances have been made in diagnosis and chemoradiotherapy, patient prognosis remains very poor. The median overall survival is less than 15 months, and the five-year survival rate is only 9.8%^7^. Therefore, the identification and development of new therapeutic agents are needed for glioma treatment.

Demethylzeylasteral (T-96) is a triterpene that is extracted from Tripterygium wilfordii Hook F (TwHF). T-96 has been shown to improve lupus nephritis in mice by inhibiting the activation of NF-κB and reducing downstream pro-inflammatory mediators, such as TNF-α, COX-2 and ICAM-1^30^. It has been reported that T-96 has the potential to inhibit tumour cell proliferation and migration^31^. A previous report showed that T-96 inhibits tumour cell migration by downregulating urokinase-type plasminogen activator^31^. Our previous studies suggested that T-96 could inhibit melanoma cell proliferation and induce apoptosis by suppressing the expression of MCL1^33^. However, the effect of T-96 on glioma remains unclear.

Triptolide, another anti-cancer compound extracted from TwHF, can induce apoptosis depending on the status of p53 in gastric cancer^38^. Herein, apoptosis was not observed in either the p53 wild-type (U-87) or mutant (LN-229) cells. These results indicated that T-96, unlike triptolide, does not induce apoptosis even in glioma cells with functional p53. Furthermore, this might also suggest that T96 induces growth arrest in both gliomas with functional p53 and those with deficient p53. Cell proliferation and differentiation show a remarkable inverse relationship, and stimuli that promote differentiation may trigger pathways that induce cell cycle arrest at G0/G1. Herein, the expression of β3-Tubulin (a neuron-specific marker) and GFAP (a glioma marker) were not changed significantly. These results clearly demonstrate that T-96 has a direct effect on proliferation.

T-96 downregulates the expression of genes from a number of DNA repair pathways, notably mismatch repair, homologous recombination, Fanconi anaemia, base excision repair and nucleotide excision repair. This suggests that T-96 might have a chemo and radiosensitizing effect in glioma cells, which may increase the efficiency of therapies currently uesed against glioma (radiotherapy in combination with TMZ). Preliminary research on these aspects was performed, and the results showed that TMZ combined with T-96 exhibited a greater inhibitory effect than TMZ alone in both LN-229 and A-172 cells (data not shown). Recent studies have shown that T-96 can enhance the chemosensitivity of pancreatic ductal adenocarcinoma cells to gemcitabine by modulating the crosstalk between apoptosis and autophagy^32^. These results indicated that T-96 is a potential therapeutic agent for cancer. However, further studies are need.

MYBL2, a nuclear protein involved in cell cycle progression, was found to be significantly downregulated after T-96 treatment. Our results suggested that MYBL2 overexpression significantly rescued T-96-induced cell growth and proliferation inhibition. The cell cycle analysis outcomes showed that MYBL2 overexpression rescued the cell cycle arrest caused by T-96. Further studies showed that the expression levels of proteins related to cell cycle and DNA replication were markedly higher in MYBL2-overexpressing cells than in control cells after treatment with T-96. These results suggest that overexpression of MYBL2 might enhance drug resistance.

MYBL2, also known as B-MYB, is a member of the MYB family of transcription factors. MYBL2 is widely expressed in all proliferating cells^39^ and is crucial for regulating the cell cycle, differentiation and apoptosis^40–42^. MYBL2 is overexpressed in various cancers, including leukaemia^43^, neuroblastoma^44^, breast cancer^45^, lung cancer^46^, colorectal cancer^47^ and hepatocellular carcinoma^48^. It has also been reported that MYBL2 expression is correlated with poor prognosis in several cancers^47,49^. By analysing the R2 database, we found that glioma patients with higher MYBL2 expression levels showed poorer prognosis (Fig. S9). Moreover, the expression level of MYBL2 was positively correlated with the classification and staging of tumours. These results indicated that MYBL2 may be a meaningful prognostic indicator of glioma and might play oncogenic roles in tumour development.

MYBL2 regulates cell cycle progression through the S and G2/M phases^50–55^. Several studies have revealed that MYBL2 regulates many cell cycle-associated genes and critical regulators, such as cyclin B1 and CDC2^56–58^. In this study, we found an association between the MYBL2 gene and the expression levels of multiple genes related to cell cycle and DNA replication in glioma patients (Table S1). It was speculated that MYBL2 might act as a master switch to control these genes.

Interestingly, exogenous MYBL2 protein was not significantly decreased after treatment with T-96 (Fig. 5g). We speculated that T-96 may act as a suppressor of MYBL2 expression at the transcriptional level. Several studies have revealed that MYBL2 is regulated by miRNAs. For example, MYBL2 has potential applications in the clinical diagnosis of acute myeloid leukaemia and is associated with the microRNA-30 family^59^. In cervical carcinoma cells, miR-29 and miR-30 regulate the expression of MYBL2 by binding to its 3’UTR, and these miRNAs can inhibit cellular DNA synthesis and play essential roles in Rb-driven cellular senescence^34^. During colon epithelial cell maturation, MYBL2 is negatively regulated by miR-365^60^. Here, miR-30e-5p, which can target MYBL2^34^, was significantly upregulated after treatment with T-96 compared with controls in both LN-229 and A-172 cells. Correspondingly, the expression of MYBL2 was significantly decreased. Our results demonstrated that inhibiting miR-30e-5p expression by the antagomir reversed the inhibitory effect of T-96 on MYBL2 expression and rescued T-96-induced inhibition. These results indicated that T-96 might silence MYBL2 expression by upregulating miR-30e-5p and then inhibiting human glioma cell growth (Fig. 7j).

The current study presents a promising novel agent with anti-tumour activity against human glioma cells in vitro and in vivo. Our results suggested that T-96 significantly inhibited cell growth and induced cell cycle arrest in the G1 phase but did not induce apoptosis or senescence in glioma cells. The expression of miR-30e-5p was noticeably increased after treatment with T-96, and its target gene MYBL2, which is involved in cell cycle progression, was significantly reduced in synchrony. Overexpression of MYBL2 partially rescued the T-96-induced inhibition of cell growth and proliferation. Moreover, an antagomir effectively inhibited the expression of miR-30e-5p and partially rescued the T-96-induced inhibition of cell growth and proliferation, accompanied by reversal of MYBL2 expression. More important, T-96 effectively upregulated miR-30e-5p expression, downregulated MYBL2 expression and inhibited tumour growth in a mouse tumour model developed with LN-229 cells. In conclusion, our work demonstrated that T-96 may inhibit glioma cell growth by regulating the miR-30e-5p/MYBL2 axis in vitro and in vivo. Thus, T-96 may be a promising therapeutic agent for glioma treatment.

## Materials and Methods

### Cell culture

The human glioma cell lines LN-229, U-87, A-172, U-251 and U-118 and the human embryonic renal cell line 293FT were purchased from the American Type Culture Collection (ATCC, USA) and stored in our laboratory. LN-229 cells express wild-type PTEN and a p53 gene mutation, while U-87 cells havewild-type p53 with a PTEN mutation. The A-172, U-251 and U-118 cell lines all have the p53 and PTEN mutation. Glioma cell lines were cultured in Dulbecco’s modified Eagle’s medium (DMEM, Gibco, New York, NY, USA) supplemented with 10% foetal bovine serum (FBS, Gibco) and 1% penicillin and streptomycin (P/S, Invitrogen, Carlsbad, CA, USA). 293FT cells were cultured in DMEM containing 0.5 mg/mL G418 (Invitrogen), 1 mM sodium pyruvate (Invitrogen), 0.1 mM non-essential amino acids (Invitrogen), and 4 mM L-glutamine (Invitrogen). The 293FT transfection medium did not contain P/S or G418. All cells were cultured at 37 °C in a humidified atmosphere containing 5% CO_2_.

### Reagents and antibodies

Demethylzeylasteral was purchased from MUST BIO-TECHNOLOGY (Cheng Du, China) and dissolved in DMSO. Anti-Cyclin D1 (2922), anti-CDK4 (12790), anti-CDK6 (2546), anti-GFAP (12389), anti-t3-Tubulin (5666), anti-E-cadherin (14472), anti-Vimentin (5741) and anti-MCM2 (3619) antibodies were obtained from Cell Signalling Technology (CST, Boston, MA, USA). Anti-5’-bromo-2-deoxyuridine (BrdU) (ab6326), anti-CDC6 (ab109315), Anti-CDT1 (ab202067), anti-CDC45L (ab108350), anti-ORC6 (ab128923) and anti-MCM3 (ab88686) antibodies were purchased from abcam (Cambridge, MA, USA). Anti-MYBL2 antibody (bs-5960R) was obtained from Bioss (Beijing, China). Anti-Flag tag antibody (AF519), anti-Tubulin antibody, RIPA Lysis Buffer, Senescence β-Galactosidase Staining Kit, and 3, 3’-diaminobenzidine (DAB) were obtained from Beyotime (Shanghai, China). 5-Bromo-2-deoxyuridine (BrdU), 3-[4, -5-dimethylthiazol-2-yl]-2, -5-diphenyltetrazolium bromide (MTT) (M5655) and dimethyl sulfoxide (DMSO, D5879) were obtained from Sigma-Aldrich (St. Louis, MO, USA). 4', -6-Diamidino-2-phenylindole (DAPI), Alexa Fluor 488 goat anti-rabbit IgG (H+L) and puromycin (A1113803) were purchased from Life Technologies (Life, New York, NY, USA). The transfection reagent NDE3000 was obtained from Western Biological Technology Co., Ltd., Chongqing, China. HRP goat anti-mouse and goat anti-rabbit antibodies were purchased from Beyotime (Shanghai, China). Ki67 antibody (550609), Annexin V-FITC Apoptosis Detection Kit and propidium iodide (PI) were obtained from BD Pharmingen (BD, San Jose, CA, USA). Antagomir miR-30e-5p (UGUAAACAUCCUUGACUGGAAG) was purchased from RiboBio (Guang Zhou, China).

### Cell viability and proliferation assays

Cell proliferation was determined with MTT assays, as previously reported^35,61^. Briefly, 1000 cells/well were plated onto 96-well plates and allowed to attach overnight at 37 °C. Culture medium containing DMSO or T-96 was added to each well, and the cells were cultured at 37 °C. At the indicated time, 20 μL MTT (Sigma, USA, 5 μg/ml) was added to each well; the cells were incubated at 37 °C for 2 h, and the formazan complex was removed with DMSO. The absorbance was measured at a wavelength of 560 nm using a microplate reader (Thermo Fisher, Waltham, MA, USA).

### BrdU staining

Briefly, 10,000 cells were cultured in 24-well plates overnight at 37 °C. Culture medium containing DMSO or T-96 was added to each well, and cells were cultured at 37 °C °C for 2 days. After incubation with BrdU (Abcam, USA, 10 μg /ml) for 1 h, the cells were washed with phosphate-buffered saline (PBS) and fixed in 3.7% paraformaldehyde for 10 min. Subsequently, the cells were treated with 2 M HCl at room temperature for 10 min and then incubated at 37 °C for 15 min. After being washed three times with PBS buffer, the cells were blocked with 10% goat serum (ZSGB-Bio, Beijing, China) containing 0.5% Triton X-100 at 37 °C for 2 h. Cells were incubated with a monoclonal rat primary antibody against BrdU (1:1000) at 4 °C overnight, followed by incubation with Alexa FluorR® 488 goat anti-rat IgG secondary antibody (H + L; 1:10,000, Invitrogen). The nuclei were stained with DAPI. The percentage of BrdU staining was calculated from at least 10 microscopic fields.

### Flow cytometry

After treatment with DMSO or T-96 at 37 °C for 3 days, cells were harvested and washed with cold PBS buffer and then resuspended in 100 μL of binding buffer (BD, USA). Then, the cells were incubated with FITC-labelled Annexin V (BD, San Jose, CA, USA) and propidium iodide (PI, BD, USA) at room temperature for 15 min and analysed via flow cytometry as previously described^62^. For the cell cycle assay, cells were harvested and fixed in ice-cold 70% ethanol at 4 °C for 1 day. After being washed and resuspended in PBS buffer, the cells were incubated with PI and RNase A (Sigma Aldrich, USA) at 37 °C for 1 h. All samples were analysed using a FACS C6 (BD, USA) with Cell Quest software.

### Western blot analysis

LN-229, U-87 and A-172 glioma cell lines were harvested, washed with ice-cold PBS buffer and then suspended in RIPA lysis buffer (Beyotime, China) with phosphatase inhibitors (Sigma Aldrich, St. Louis, MO, USA) and complete protease inhibitor cocktail (Roche). Protein concentrations were detected with a BCA protein assay kit (Beyotime Biotech, China). Then, 30 μg of lysate was subjected to 10% SDS-PAGE and transferred onto a PVDF membrane (Millipore, USA). After being blocked with 5% bovine serum albumin (BSA) in TBST buffer at room temperature for 1–4 h, the PVDF membranes were gently incubated with specific primary antibodies against human tubulin (1:1000), CDK2 (1:1000), CDK6 (1:1000), cyclin D1 (1:1000), GFAP (1:1000), β3-tubulin (1:1000), E-cadherin (1:1 000), vimentin (1:1000), CDC6 (1: 10,000), CDT1 (1:10,000), CDC45 (1:10,000), MCM2 (1:1000), MCM3 (1:10,000), ORC6 (1:1 000), MYBL2 (1:100) and Flag tag (1:1000) at 4 °C overnight. After a wash with TBST buffer, the PVDF membranes were incubated with horseradish peroxidase (HRP)-labelled goat anti-mouse IgG (H + L) (A0216, 1:10,000) or goat anti-rabbit IgG (H + L) (A0208, 1:10,000) at room temperature for 2 h. Protein bands were visualised with SuperSignal West Femto Maximum Sensitivity Substrate (Thermo Fisher, Waltham, MA, USA), and luminescence images were analysed with a Western blot detection instrument (Clinx Science, Shanghai, China).

### Transfection and infection

Human full-length MYBL2 cDNA (NM_002466.3) was downloaded from the National Center for Biotechnology Information (NCBI), and the full-length coding sequence of MYBL2 was amplified by PCR and ligated into a PCDH-CMV-Flag-MCS-EF1-puro vector by Wuhan GeneCreate Biological Engineering Co, Ltd (Wuhan, China). Lentiviral production and infection and establishment of stable cell lines overexpressing the MYBL2 gene were conducted as previously described^63^. Cells were transiently transfected with the miR-30e-5p antagomir or anti-miRNA inhibitor negative control in accordance with the reagent instructions.

### RNA sequencing, data analysis and qRT-PCR

After treatment with DMSO or T-96 at 37 °C for 2 days, cells were harvested, and total RNA was isolated from LN-229 and U-87 cells using Trizol reagent according to the manufacturer’s instructions. Partial RNA samples were then submitted to BIOMARKER (Beijing, China) for transcriptome sequencing and analysis. RNA-seq libraries were prepared with 250 ng of RNA using a TruSeq stranded mRNA sample preparation kit (Illumina Inc., USA) according to the instruction and operation manual. The libraries were then sequenced using a HiSeq platform (Illumina, San Diego, CA, USA) on a 150-bp paired-end run. All raw data are uploaded to Sequence Read Archive (SRA, accession: PRJNA487962). A *p*-value cut-off for false discovery rate (FDR) of 0.05 and a minimum 2-fold change in expression were uesed to screen for differentially expressed genes. Differentially expressed genes were validated by quantitative real-time PCR (qPCR). Total RNA was reverse-transcribed to cDNA using M-MLV reverse transcriptase (Promega), and qPCR was performed as described in our previous studies^64,65^. The primers used in this study are listed in Table S2. *GAPDH* was used as the internal control. Relative mRNA expression levels were calculated using the 2^−ΔΔCT^ method. The expression of miR-30e-5p was determined by using a miRNA qRT-PCR assay, as described in previous study^66^.

### Soft agar colony formation assay

The effect of T-96 on the colony formation ability of LN-229 and U-87 cells was determined with a soft agar assay. Briefly, 1.5 mL of DMEM medium containing 0.6% agarose was gently added to each well of a six-well culture plate, and then, 1 mL of DMEM containing 0.3% agarose, 1000 cells and T-96 at different concentration gradients was added to the top of the solidified bottom layer. After 2 to 3 weeks of culture, the cells were stained with MTT, and photographs were taken with a digital camera.

### Animal studies

Five-week-old female nude mice were used in these experiments, as previously described^35^. Animal studies were performed in accordance with the Guidelines of the Institute for Laboratory Animal Research, Southwest University (Chongqing, China). Glioma LN-229 cells (1 × 10^6^ cells) in 100 μL of PBS were subcutaneously injected into both flanks of each mouse. When the tumours reached a certain volume, T-96 (30 mg/kg) was injected once every two days. DMSO injections were administered to control mice, and tumour volume was measured every 2 days. Two days after the sixth injection, the mice were killed, and tumours were collected and analysed.

In orthotopic implantation assays, glioma LN-229 cells (1 × 10^5^ cells) were intracranially injected slowly into the brains of each mouse. After one week, the mice were intraperitoneally injected with T-96 (30 mg/kg) or an equivalent amount of DMSO every other day for 20 days. Then, the brains were collected, processed and analysed as described previously^67^.

### Statistical analysis

All experiments were carried out with three technical and biological replicates. All the results acquired in this study are presented as the means ± standard deviation (SD). Differences between means were determined via unpaired Student’s *t*-tests, and *p* < 0.05 was considered statistically significant.

## Electronic supplementary material

T-96 inhibited glioma cell growth in vitro

T-96 inhibited glioma cell growth but not through apoptosis

T-96 inhibited glioma cell growth but not through senescence in glioma cells

The expression of genes related to the cell cycle after treatment with 10 μM T-96 in LN-229 (a) and U-87 (b) cells with 10 μM T-96

MYBL2 is widely expressed in glioma cells

Quantitative real-time PCR assays were used to detect the expression of MYBL2 in LN-229, U-87, and A-172 cells after treatment with T-96

After cell treatment with T-96, quantitative real-time PCR assays were used to detect the expression of all the miRNAs that could theoretically target MYBL2

The weight of mice was measured after DMSO or T-96 treatment

High expression of MYBL2 was correlated with poor prognosis

Relationships between MYBL2 mRNA levels and genes related to the cell cycle and DNA replication in human glioma patients

Primers used for real-time quantitative PCR analysis

Supplementary figure legends
