# Supplementary figures and images for: Demethylzeylasteral inhibits glioma growth by regulating the miR-30e-5p/MYBL2 axis
Source: Cell Death Dis. 2018 Oct 10;9(10):1035. doi: 10.1038/s41419-018-1086-8 (PMC6180101; doi:10.1038/s41419-018-1086-8)

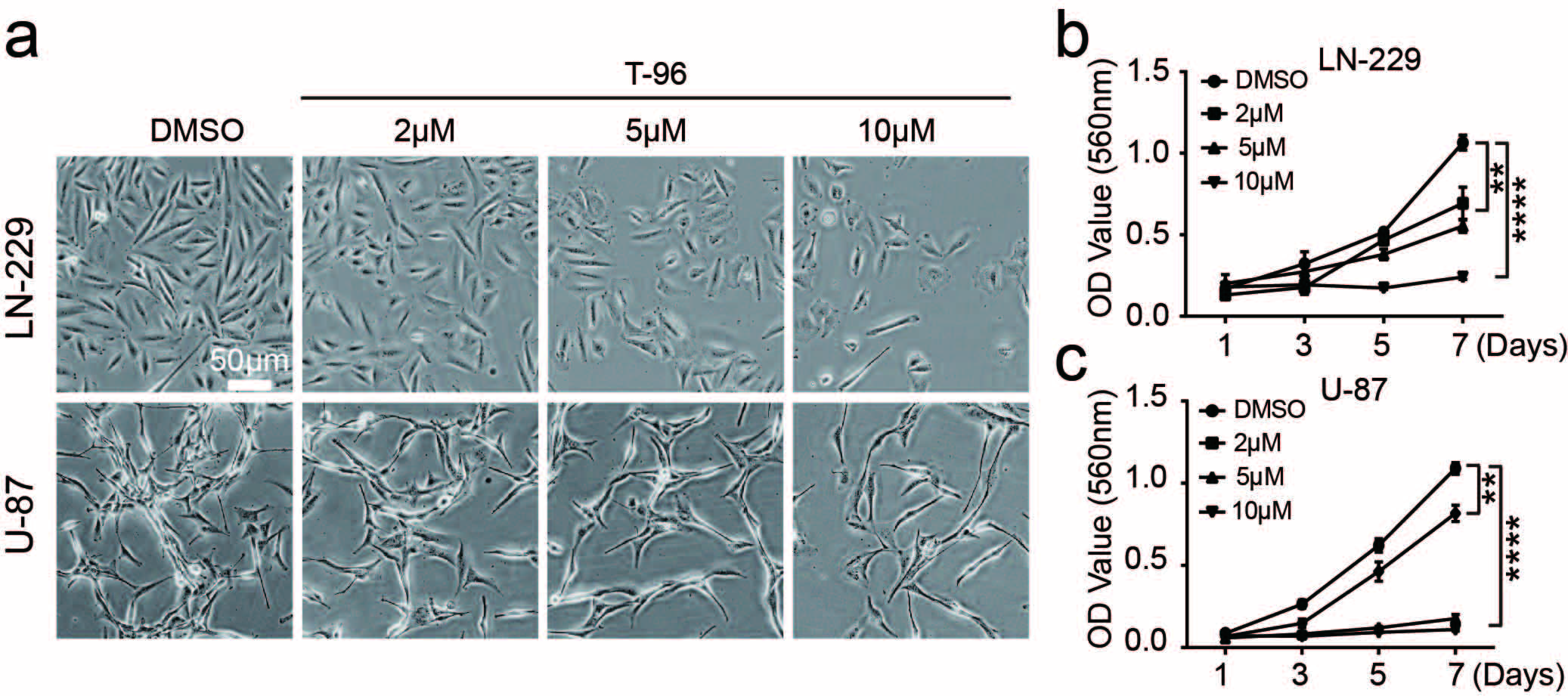

Supplement: Supplementary file 1 — T-96 inhibited glioma cell growth in vitro [file 41419_2018_1086_MOESM1_ESM.jpg]

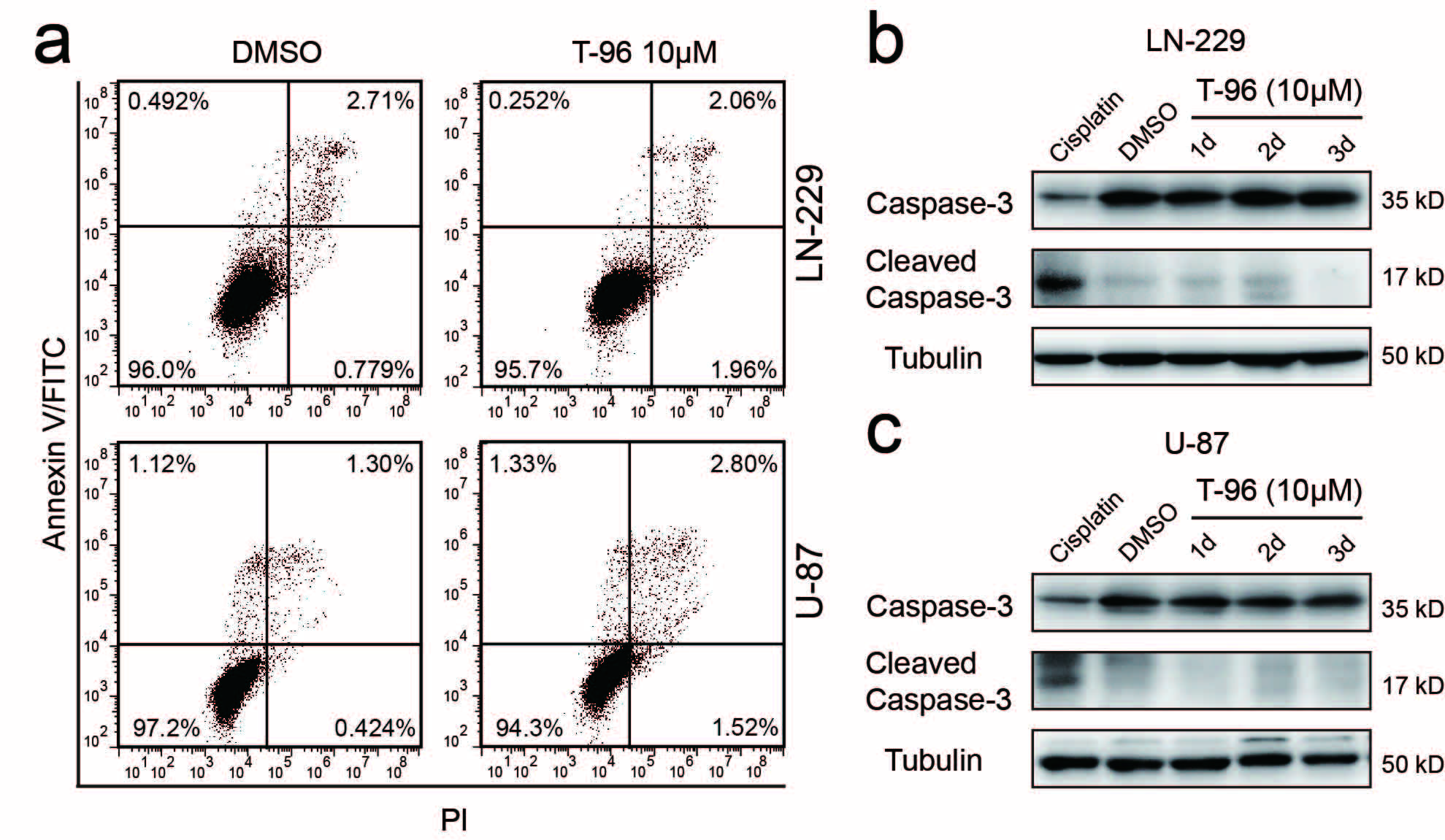

Supplement: Supplementary file 2 — T-96 inhibited glioma cell growth but not through apoptosis [file 41419_2018_1086_MOESM2_ESM.jpg]

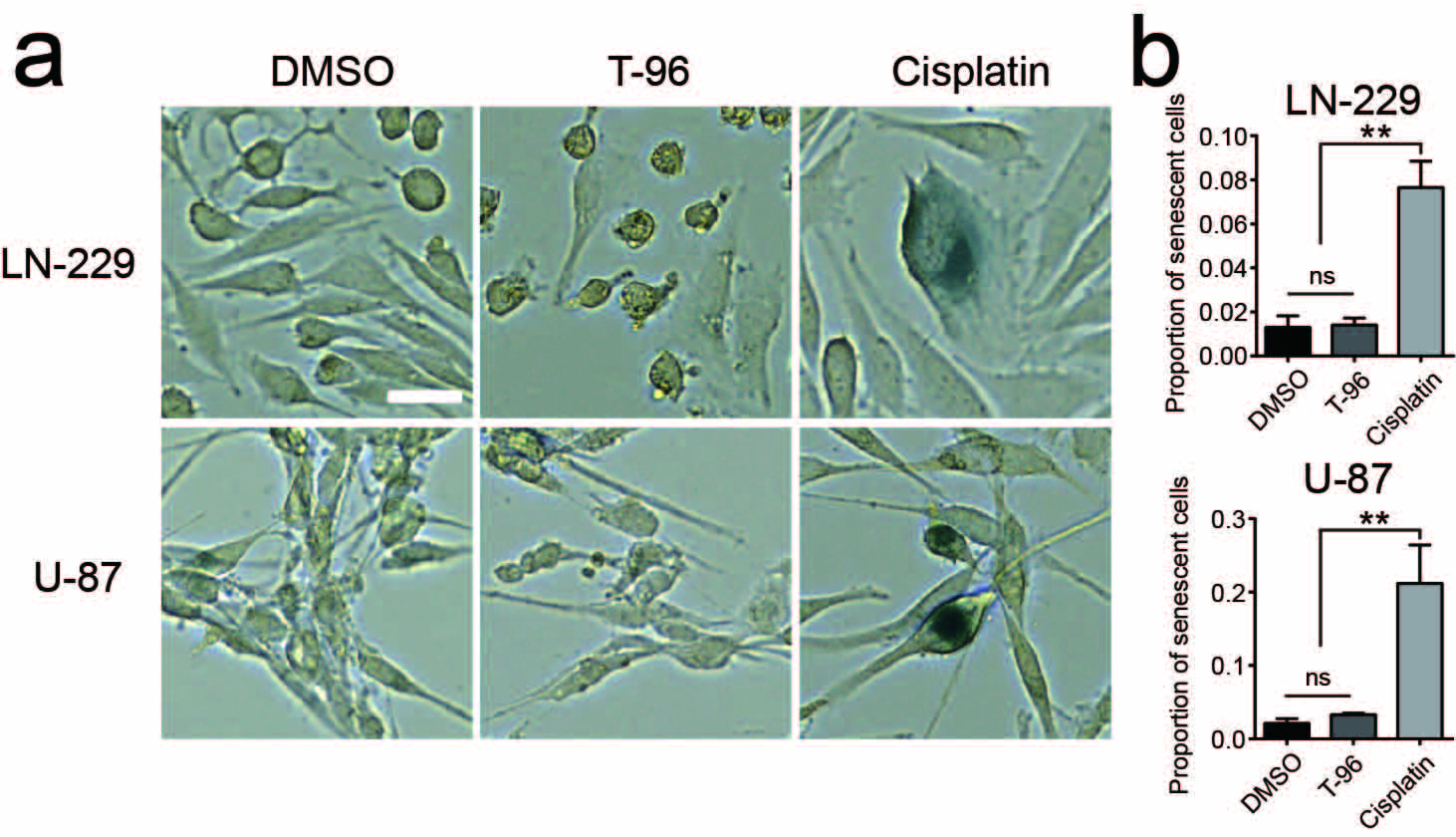

Supplement: Supplementary file 3 — T-96 inhibited glioma cell growth but not through senescence in glioma cells [file 41419_2018_1086_MOESM3_ESM.jpg]

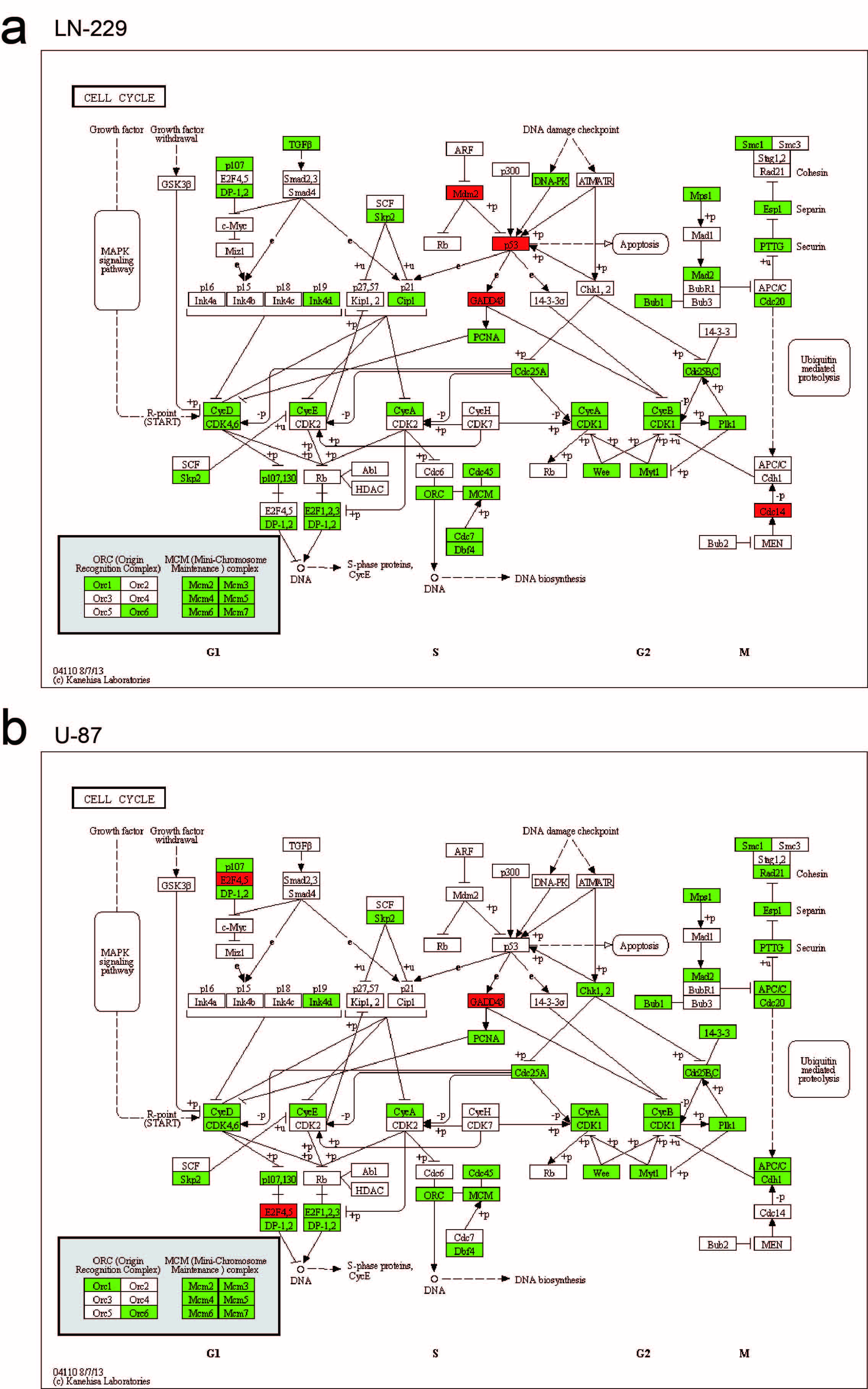

Supplement: Supplementary file 4 — The expression of genes related to the cell cycle after treatment with 10 μM T-96 in LN-229 (a) and U-87 (b) cells with 10 μM T-96 [file 41419_2018_1086_MOESM4_ESM.jpg]

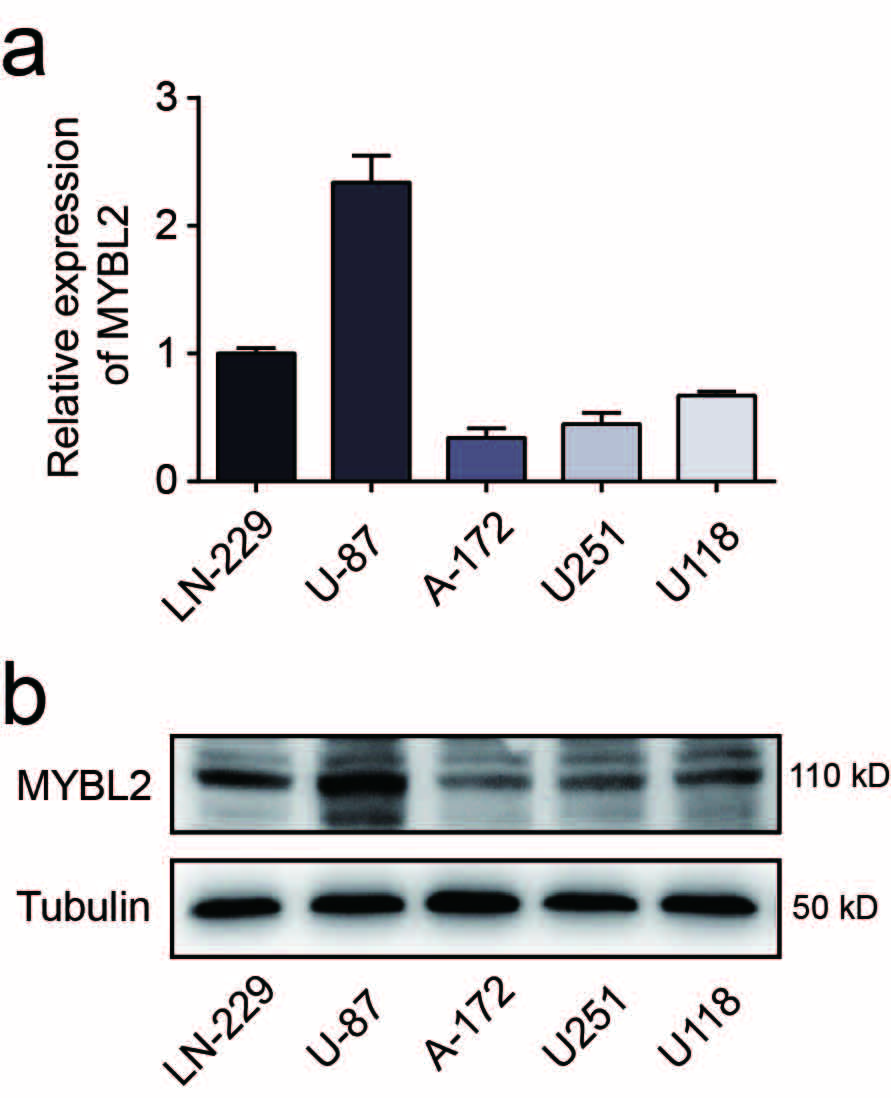

Supplement: Supplementary file 5 — MYBL2 is widely expressed in glioma cells [file 41419_2018_1086_MOESM5_ESM.jpg]

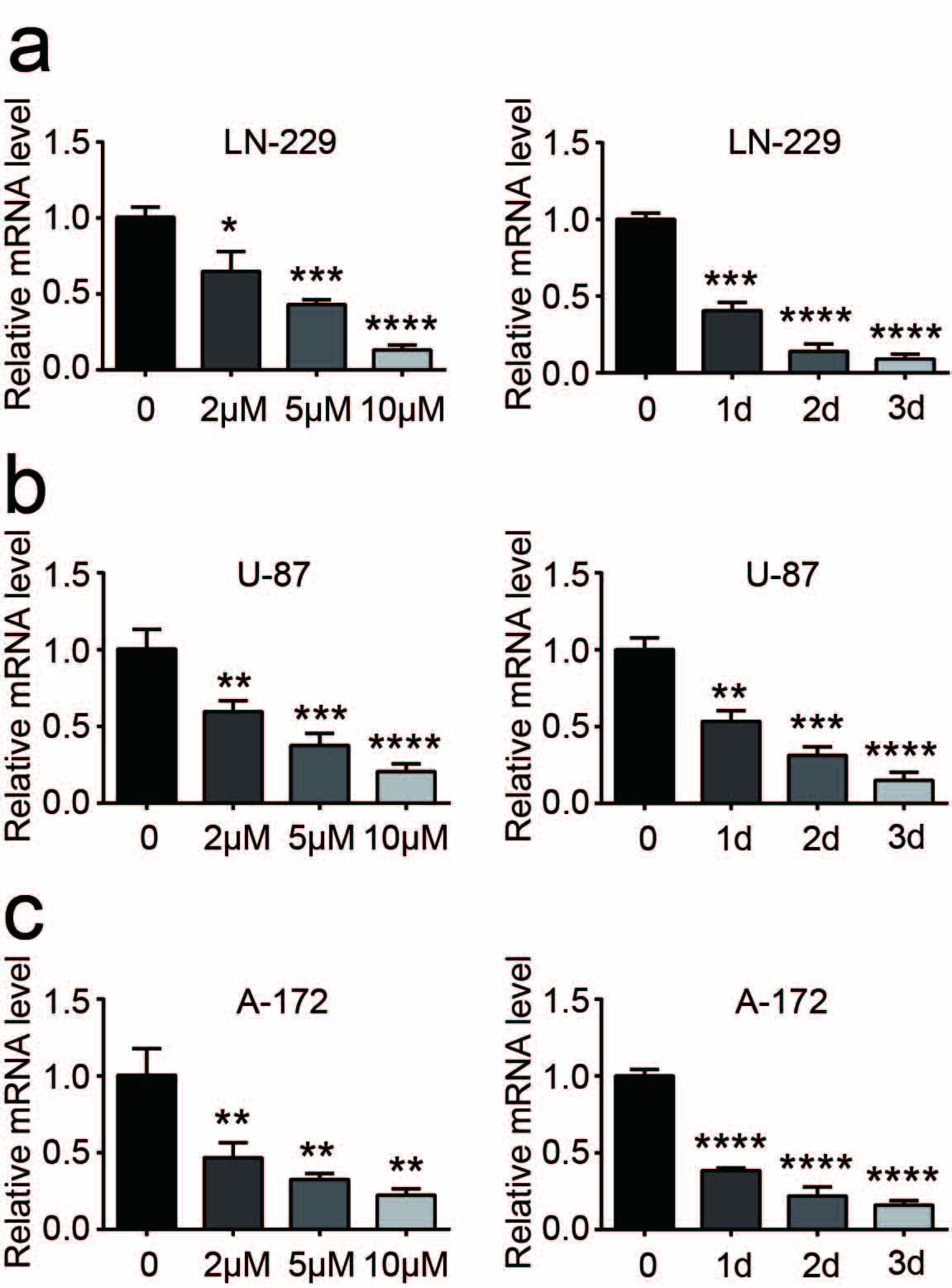

Supplement: Supplementary file 6 — Quantitative real-time PCR assays were used to detect the expression of MYBL2 in LN-229, U-87, and A-172 cells after treatment with T-96 [file 41419_2018_1086_MOESM6_ESM.jpg]

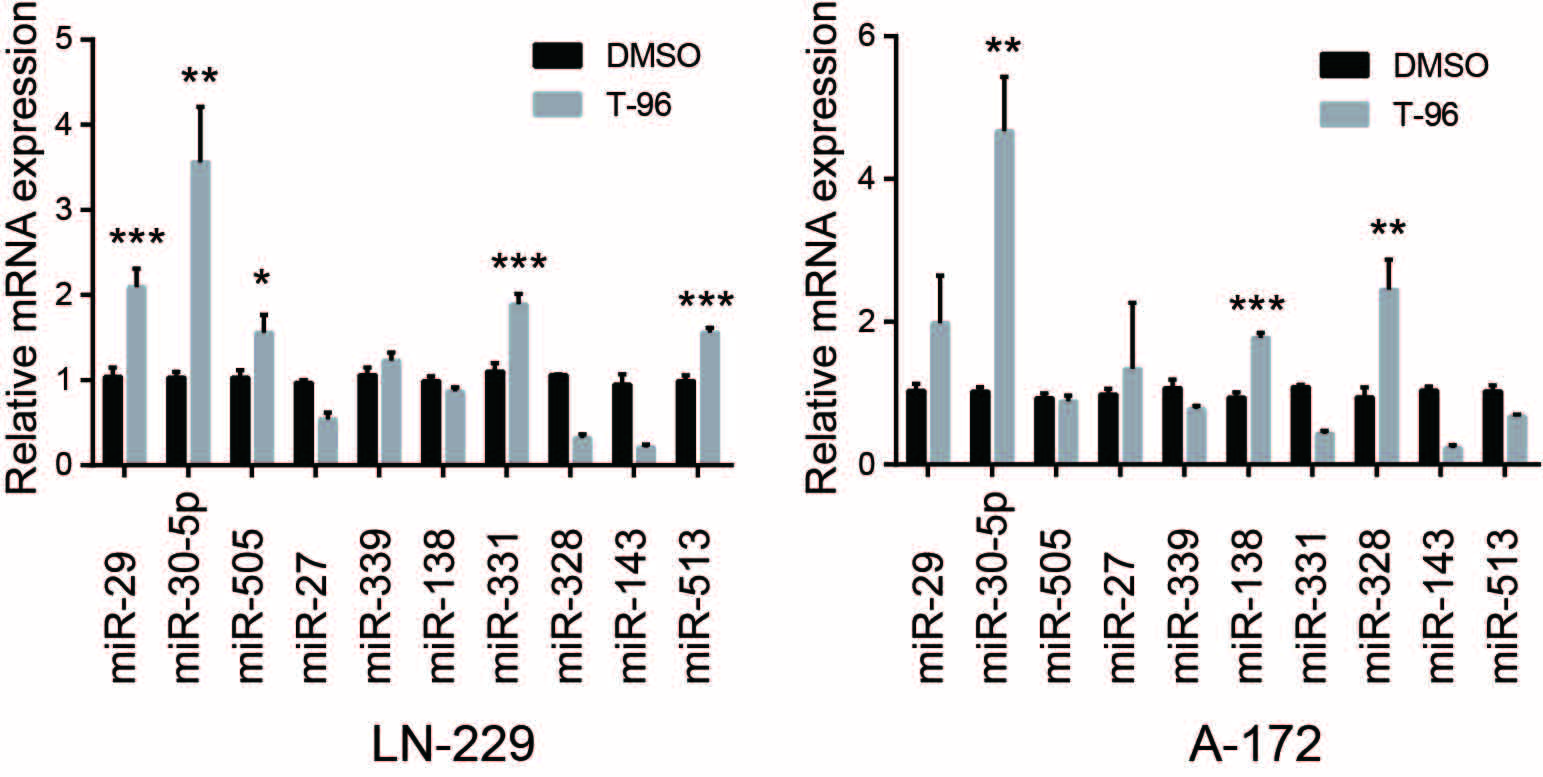

Supplement: Supplementary file 7 — After cell treatment with T-96, quantitative real-time PCR assays were used to detect the expression of all the miRNAs that could theoretically target MYBL2 [file 41419_2018_1086_MOESM7_ESM.jpg]

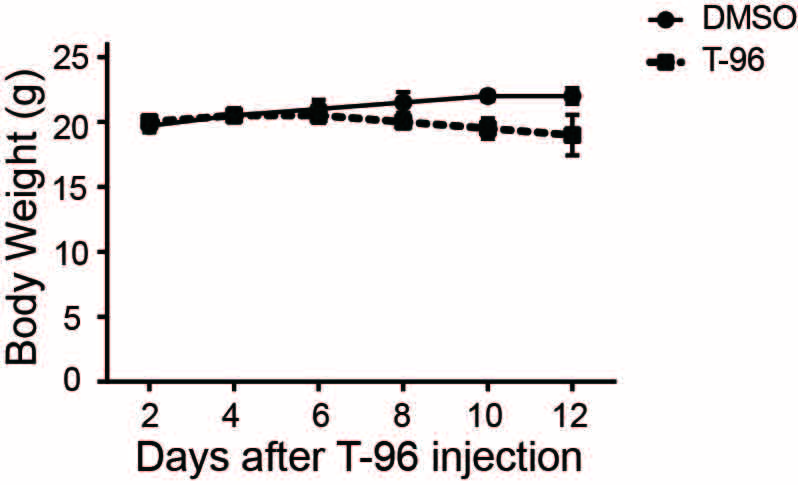

Supplement: Supplementary file 8 — The weight of mice was measured after DMSO or T-96 treatment [file 41419_2018_1086_MOESM8_ESM.jpg]

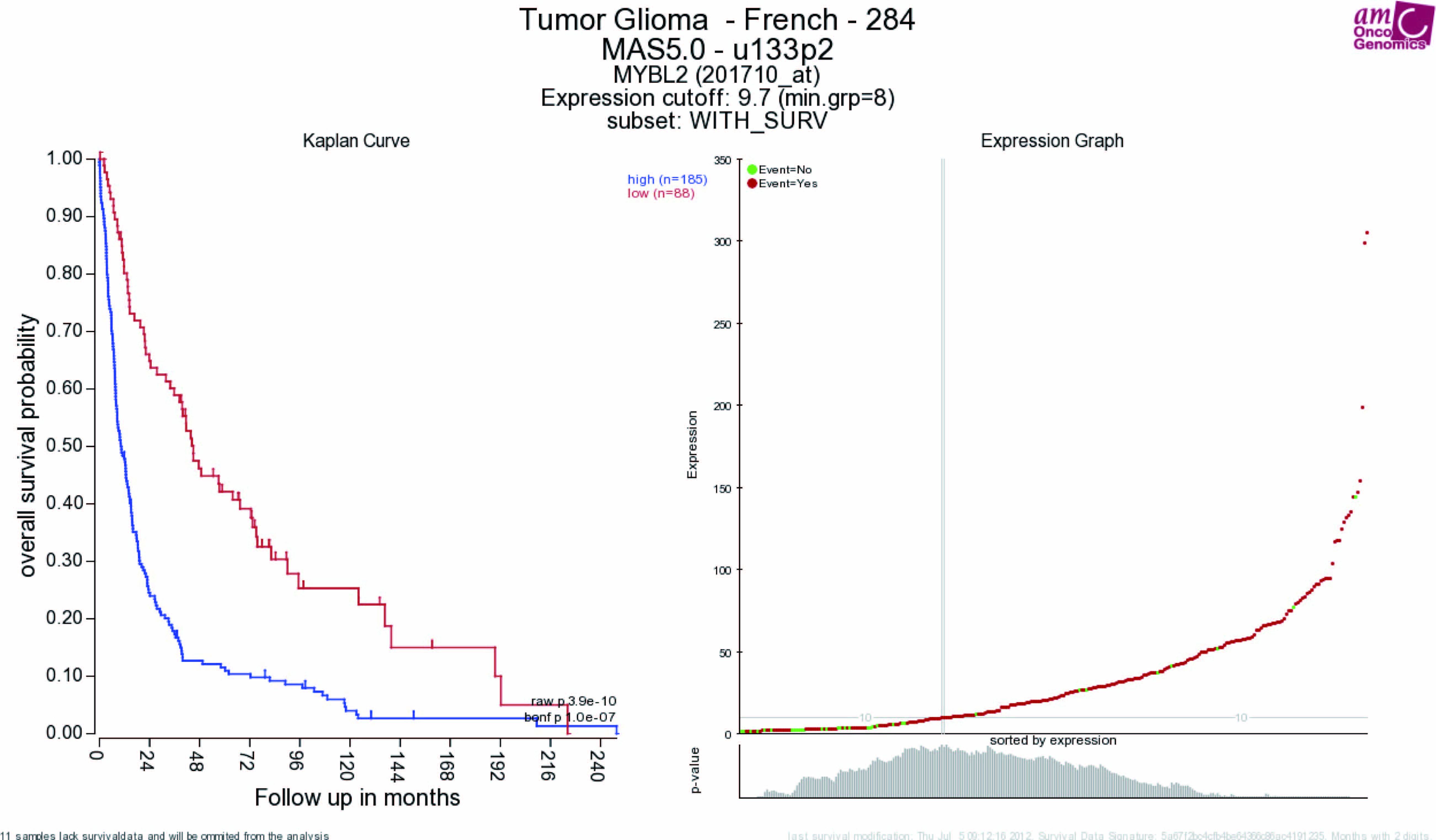

Supplement: Supplementary file 9 — High expression of MYBL2 was correlated with poor prognosis [file 41419_2018_1086_MOESM9_ESM.jpg]
