# Supplementary material for: Demethylzeylasteral inhibits glioma growth by regulating the miR-30e-5p/MYBL2 axis
Source: Cell Death Dis. 2018 Oct 10;9(10):1035. doi: 10.1038/s41419-018-1086-8 (PMC6180101; doi:10.1038/s41419-018-1086-8)
Supplement: Supplementary file 12 — Supplementary figure legends [file 41419_2018_1086_MOESM12_ESM.docx]

Figure S1 T-96 inhibited glioma cell growth *in vitro*. (a) Cell morphology of LN-229 and U-87 cells after treatment with DMSO or the indicated concentration of T-96 for 2 days; scale bar = 50 μm. (b, c) Cell growth in cells treated with T-96 at the indicated times or concentrations was monitored using MTT assays. All data were analysed using unpaired Student’s t-tests and are shown as the means ± SD. **p < 0.01, ****p < 0.0001.

Figure S2 T-96 inhibited glioma cell growth but not through apoptosis. (a) LN-229 and U-87 cells were treated with DMSO or 10 μM T-96 for 3 days, and apoptosis was analysed by Annexin V/PI staining using flow cytometry. (b, c) Western blot assays were performed to detect the expression of cleaved caspase-3 after T-96 treatment.

Figure S3 T-96 inhibited glioma cell growth but not through senescence. (a) LN-229 and U-87 cells were treated with DMSO or 10 μM T-96 for 2 days, and senescence was analysed using a senescence β-galactosidase staining kit. Cisplatin (10 μM) was used as the positive control. Scale bar = 50 μm. (b) The histogram demonstrates the quantification results for the number of β-galactosidase staining-positive LN-229 and U-87 cells. All data were analysed using unpaired Student’s t-tests and are shown as the means ± SD. **p < 0.01.

Figure S4 The expression of genes related to the cell cycle after treatment LN-229 (a) and U-87 (b) cells with 10 μM T-96. The green and red squares represent the down- and upregulated genes, respectively.

Figure S5 MYBL2 is widely expressed in glioma cells. Real-time PCR (a) and Western blotting (b) were used to detect the expression of MYBL2 in different glioma cell lines, including LN-229, U-87, A-172, U251, and U-118 cells.

Figure S6 Quantitative real-time PCR assays were used to detect the expression of MYBL2 in LN-229, U-87, and A-172 cells after treatment with T-96. All data were analysed using unpaired Student’s t-test and are shown as the means ± SD. *p < 0.05, **p < 0.01, ***p < 0.001, ****p < 0.0001.

Figure S7 After cell treatment with T-96, quantitative real-time PCR assays were used to detect the expression of all the miRNAs that could theoretically target MYBL2. All data were analysed using unpaired Student’s t-tests and are shown as the means ± SD. *p < 0.05, **p < 0.01, ***p < 0.001.

Figure S8 The weight of mice was measured after DMSO or T-96 treatment.

Figure S9 High expression of MYBL2 was correlated with poor prognosis. Kaplan-Meier analysis of the progression-free survival of patients in the glioma dataset.

Table S1 Relationships between MYBL2 mRNA levels and genes related to the cell cycle and DNA replication in human glioma patients.

Table S2 Primers used for real-time quantitative PCR analysis.
